# Found in Complexity, Lost in Fragmentation: Putting Soil Degradation in a Landscape Ecology Perspective

**DOI:** 10.3390/ijerph19052710

**Published:** 2022-02-25

**Authors:** Rares Halbac-Cotoara-Zamfir, Gloria Polinesi, Francesco Chelli, Luca Salvati, Leonardo Bianchini, Alvaro Marucci, Andrea Colantoni

**Affiliations:** 1 Department of Overland Communication Ways, Foundation and Cadastral Survey, Politehnica University of Timisoara, 1A I. Curea Street, 300224 Timisoara, Romania; rares.halbac-cotoara-zamfir@upt.ro; 2 Department of Mathematics, Computer Science and Economics, University of Basilicata, 85100 Potenza, Italy; glopol@hotmail.it; 3 Department of Economics and Law, University of Macerata, Via Armaroli 43, 62100 Macerata, Italy; luca.salvati@unimc.it; 4 Department of Agricultural and Forestry Sciences (DAFNE), University of Tuscia, Via San Camillo de Lellis, 01100 Viterbo, Italy; l.bianchini@unitus.it (L.B.); marucci@unitus.it (A.M.); colantoni@unitus.it (A.C.)

**Keywords:** ecological metrics, desertification risk, agricultural mechanization, land-use change, multivariate analysis, Europe

## Abstract

The United Nations Convention to Combat Desertification (UNCCD) assumes spatial disparities in land resources as a key driver of soil degradation and early desertification processes all over the world. Although regional divides in soil quality have been frequently observed in Mediterranean-type ecosystems, the impact of landscape configuration on the spatial distribution of sensitive soils was poorly investigated in Southern Europe, an affected region *sensu* UNCCD. Our study proposes a spatially explicit analysis of 16 ecological metrics (namely, patch size and shape, fragmentation, interspersion, and juxtaposition) applied to three classes of a landscape with different levels of exposure to land degradation (‘non-affected’, ‘fragile’, and ‘critical’). Land classification was based on the Environmentally Sensitive Area Index (ESAI) calculated for Italy at 3 time points along a 50-year period (1960, 1990, 2010). Ecological metrics were calculated at both landscape and class scale and summarized for each Italian province—a relevant policy scale for the Italian National Action Plan (NAP) to combat desertification. With the mean level of soil sensitivity rising over time almost everywhere in Italy, ‘non-affected’ land became more fragmented, the number of ‘fragile’ and ‘critical’ patches increased significantly, and the average patch size of both classes followed the same trend. Such dynamics resulted in intrinsically disordered landscapes, with (i) larger (and widely connected) ‘critical’ land patches, (ii) spatially diffused and convoluted ‘fragile’ land patches, and (iii) a more interspersed and heterogeneous matrix of ‘non affected’ land. Based on these results, we discussed the effects of increasing numbers and sizes of ‘critical’ patches in terms of land degradation. A sudden expansion of ‘critical’ land may determine negative environmental consequences since (i) the increasing number of these patches may trigger desertification risk and (ii) the buffering effect of neighboring, non-affected land is supposed to be less efficient, and this contains a downward spiral toward land degradation less effectively. Policy strategies proposed in the NAPs of affected countries are required to account more explicitly on the intrinsic, spatio-temporal evolution of ‘critical’ land patches in affected regions.

## 1. Introduction

Biophysical dynamics and socioeconomic forces leveraged land degradation, causing an evident reduction in soil fertility and land productivity [1,2,3]. The reduction of soil’s potential for both agricultural and natural productions is one of the main factors at the base of land degradation, a key environmental issue worldwide [4]. At the same time, population growth, unrested economic development, and climate change, in turn, leveraged land degradation [5]. The Mediterranean region is considered a paradigmatic hotspot of soil degradation thanks to the coexistence of multiple phenomena such as erosion, salinization, compaction, sealing, and contamination at close locations [6]. A joint set of factors including population expansion, agricultural intensification, industrialization, and urban sprawl—in ecological contexts featuring climate aridity and more recurrent droughts than in the past—have largely shaped such processes [7,8,9,10]. Altogether, these forces were demonstrated to exert negative effects on soil quality [11,12,13].

The negative impact of a given factor on both sensitive and insensitive soils is assumed to be spatially ‘neutral’ (i.e., causing an increase in the level of sensitivity across a sufficiently broad area) or spatially ‘asymmetric’ (e.g., determining a rise in the degree of sensitivity specifically in already affected areas, or in non-affected land). In this perspective, soil degradation processes have been subjected to many changes and create multiple disturbances, due to the abovementioned causes, particularly over-extraction of underground water, which has led to plant degradation and soil crusting, as well as overgrazing in dryland and the increased impact of mechanization on a larger scale, expanding tillage, and resulting in large areas where steppe vegetation is being destroyed [14,15,16]. For instance, the choice of specific agricultural machines is critical to reduce land sensitivity and to optimize the productive process in terms of sustainability and resilience [17].

With soil sensitivity to degradation becoming a dynamic attribute of both natural and anthropogenic ecosystems [18], a high-resolution, diachronic assessment of landscape transformations may provide a basic knowledge to delineate (and interpret) the relationship between landscape structure and desertification risk [19,20,21]. In these regards, landscape fragmentation is taken as a proxy of soil sensitivity to degradation [22]. While being characteristic of a given ecosystem’s state, land fragmentation reflects endogenous and exogenous shocks including climate change and human pressure [23]. However, relatively few studies have investigated these issues in a specific soil degradation perspective [24]. Assuming vegetation cover as a good indicator of ecosystem conservation in dry areas, earlier studies document a negative relationship between plant cover and soil erosion [7]. Sun et al. (2007) demonstrated that habitat connectivity is a proxy of desertification risk [25]. At the same time, Alados et al. (2004) demonstrated how factors influencing dominant plant cover decrease the underlying biodiversity in communities affected by soil degradation and landscape fragmentation [8,26]. Habitat fragmentation is in turn reflective of the most important consequences of land-use at the landscape level, exerting severe consequences on natural ecosystems and ecological processes [9]. These factors may impact population and community dynamics causing important modifications in the composition of plant cover [27]. With vegetation patches becoming more fragmented and homogeneous, soils were demonstrated to be more exposed to early desertification [28]. Whether these processes lead to soil degradation—in turn triggering complex transformations responsible for changes in landscape composition—has been occasionally explored [29].

In human-dominated landscapes, such as agricultural systems, soil degradation and landscape fragmentation depend on the specific disturbance regime, i.e., the spatio-temporal pattern of external shocks [30]. Socioeconomic traits can be considered in a broad-scale analysis, e.g., evidencing that disturbance gradients brought about by socioeconomic factors and historical elements of the landscape influence vegetation cover [31]. Topographical constraints were found to be additional factors affecting vegetation sensitivity to degradation [18]. While several studies have definitely analyzed fragmentation effects on vegetation or fragmentation causes [32], an integrative approach to fragmentation and the related socioeconomic forces is needed to clarify vegetation dynamics and enhance conservation of fragmented landscapes, where factors affecting vegetation and fragmentation processes interact [33]. Assuming soil sensitivity associated with intense landscape modifications, large-scale analyses evaluating the relationship between landscape fragmentation and the level of soil sensitivity are relatively scarce and partial [34]. A quantitative characterization of spatial patterns is a crucial step when assessing intrinsic linkages between landscape configuration and the underlying ecological processes related to soil degradation [35,36,37]. Compared with the rest of Europe, Mediterranean countries such as Spain, Greece, and Italy have a medium level of landscape fragmentation overall, with greater fragmentation in peri-urban, coastal areas [38].

Based on these premises, the working hypothesis of this study is that a given local system may undergo different patterns of soil sensitivity to degradation, depending on the various dynamics related to the process of fragmentation of the land patches exposed to different levels of degradation [39]. For this purpose, the Environmental Sensitive Area (ESA) framework was adopted here as an effective monitoring system of land degradation [40]. Assuming responses to soil degradation as based on a set of land management actions depending on the local context [41], the present study assesses the evolution of landscape metrics in Italy between 1960 and 2010, considering administrative regions as the elementary spatial domain [42]. In line with these perspectives, regional mitigation plans may promote a policy shift from driver-specific (and process specific) targets to a more comprehensive set of practical actions mixing responses adapted to the local context.

## 2. Methodology

### 2.1. Study Area

With a coastline of about 7600 km including islands, Italy is divided longitudinally by the Apennine Mountain chain; the Alps in turn separate Northern Italy from the European continent. Geographically speaking, Italy is partitioned into 3 macro-regions (North, Centre, South) with a total surface area extending 301,330 km^2^ and 3 elevation belts (23% flat land, 42% hilly areas, and 35% mountainous districts). Being located in the middle of the Mediterranean basin, the country is characterized by a relatively mild climate with dry/hot summers and temperate/wet winters. On average, the amount of precipitation increases with elevation while temperature regimes follow the reverse pattern [43]. In common with other Mediterranean countries, Italy displays a marked gap in socioeconomic development reflected in differential population density, settlement distribution, and natural resource capital between Northern and Southern regions [44].

### 2.2. Estimating Soil Sensitivity to Degradation

The Environmentally Sensitive Area (ESA) scheme was adopted here as a flexible and simplified procedure evaluating soil sensitivity to degradation intended as a (more or less generalized) condition underlying land degradation and desertification risk [20], with outcomes validated on the ground [40]. This framework produces a multi-dimensional index based on 14 elementary variables classifying soils according to different degrees of sensitivity to degradation that depends on 4 thematic domains: climate, soil, vegetation, and human pressure. Climate was described using average annual rainfall rate, aridity index, and aspect [45]. Soil was described considering depth, texture, slope, and the nature of the parent material. Vegetation was evaluated adopting plant cover, fire risk, protection offered by vegetation against soil erosion, and the degree of resistance to drought shown by vegetation, as basic descriptors of soil sensitivity. Human pressure has been finally quantified as the result of population dynamics and selected land-use change, evaluating population density, annual rate of population growth, and agricultural intensity. A detailed description of the methodology has been provided in a technical Supplementary File S1 to this study.

A scoring system was applied to all variables of this study, allowing the calculation of four quality indicators of climate (Climate Quality Index, CQI), soil (Soil Quality Index, SQI), vegetation (Vegetation Quality Index, VQI), and land management (Land Management Quality Index, MQI) that are estimated as the geometric mean of the scores assigned to each input variable. Each quality indicator ranges from 1 (the lowest contribution to soil sensitivity) to 2 (the highest contribution to soil sensitivity). The ESAI was finally estimated in each spatial unit and year as the geometric mean of the four quality indicators, resulting in a score that ranges between one (the lowest sensitivity to degradation) and two (the highest sensitivity to degradation).

Three classes of soil sensitivity were identified: (i) non-affected or (potentially affected) land (ESAI < 1.225), (ii) ‘fragile’ land (1.225 < ESAI < 1.375), and (iii) ‘critical’ land (ESAI > 1.375). Maps have been produced at 1 km^2^ pixel resolution [38]. The ESAI score at each elementary spatial unit was treated as a ratio variable ranging continuously from 1 to 2. The average ESAI score was calculated separately at 103 provinces—an administrative partition of Italy reflecting the NUTS-3 level of European Nomenclature of Territorial Statistics—and 3 years (1960, 1990, 2010). This country’s partition is consistent with the characteristics and resolution of the indicators selected; in these regards, the Italian National Action Plan (NAP) to Combat Desertification has designed administrative regions and provinces as the effective spatial units to coordinate and implement mitigation policies.

### 2.3. Logical Framework

To verify the intrinsic relationship between landscape fragmentation and soil degradation in Italy, our study evaluates landscape structure at different levels of soil sensitivity to degradation using a dashboard of 16 landscape-level metrics calculated at 3 time points (1960, 1990, 2010) on the basis of the 3 ESAI maps. Our study was articulated in two steps: First, Italian land was classified into three increasing levels of sensitivity (‘non-affected’, ‘fragile’, and ‘critical’) according to the Environmentally Sensitive Area (ESA) nomenclature, as a function of four key dimensions of land degradation (climate, soil quality, vegetation cover, and human pressure). Second, the composition, configuration, and structure of landscapes constituted of the 3 ESA sensitivity classes were studied using 16 landscape metrics at the spatial level of administrative regions, a relevant domain for environmental reporting and policy implementation. In other words, we calculated these ecological metrics on homogeneous patches constituted of spatially contiguous pixels classified within the same ESAI sensitivity class, thus forming ‘non-affected’, ‘fragile’, and ‘critical’ land patches. In these regards, ‘critical’ land patches have been considered as local ‘soil sensitivity hotspots’ possibly requiring specific mitigation measures [31], e.g., by creating new tools that evaluate negative impacts on soils (e.g., mechanization indexes to monitor soil compaction).

Landscape metrics are assumed to inform strategies contrasting soil degradation that can be implemented in Regional Action Plans (RAPs) developed in line with the guidelines of the NAP. The average ESAI score and the related landscape metrics were calculated individually for each land patch (see above) and aggregated at the spatial scale of Italian provinces. To this end, we made use of the ‘zonal statistics’ procedure developed in ArcGIS (ESRI Inc., Redwoods, CA, USA) software, elaborating a surface-weighted total (or average) of each individual indicator—including the ESAI—recorded on each elementary patch (i.e., ensemble of spatially contiguous pixels within the same sensitivity class) belonging to the spatial unit under investigation [43]. A total of 16 landscape metrics assessing patch size, fragmentation, shape, fractality, and juxtaposition (Table 1) were chosen with the aim at providing a comprehensive assessment of the spatial configuration of Italian landscapes over time based on the ESAI classification of Italy in ‘non-affected’, ‘fragile’, and ‘critical’ land [46]. These metrics were derived from the above-mentioned ESAI raster maps using simple computational tools from ArcGIS and ‘Patch Analyst’ packages at the provincial scale [47].

### 2.4. Statistical Analysis

A Multiway Factor Analysis (MFA)—an extension of Principal Component Analysis (PCA) and traditional Factor Analysis working on three-dimensional data matrices—was carried out at the provincial scale considering together 17 variables (16 landscape metrics and the average ESAI score) for 3 observation years (1960, 1990, 2010). MFA was adopted with the aim at removing (or, at least, containing) the redundancy among metrics, identifying few relevant and independent dimensions with characteristic metrics associated with sensitive soils to degradation in Italy [31]. In other words, this technique illustrated the spatial structure of the multivariate relationship between land metrics and the degree of soil sensitivity [48]. Axes with eigenvalue >1 were identified and evaluated considering together the position of loadings (metrics) and scores (provinces). The statistical distribution of the metric’s loadings on the selected axes was represented in a table comparing the three years of investigation. The spatial distribution of province’s scores on the most relevant axes was illustrated through maps and compared with the spatial distribution of the average ESAI score at the same geographical scale. Metrics were also separately calculated at the class scale for ‘non-affected’, ‘fragile’, and ‘critical’ land. Considering all the metrics presented above (except for landscape diversity metrics, which were calculated only at the landscape scale), a non-parametric hypothesis testing was developed with the aim of verifying the intrinsic differences between the three classes mentioned above separately for each metric. A Kruskal–Wallis one-way Analysis of Variance was adopted to fill this objective, testing significant differences at *p* < 0.05 after Bonferroni’s correction for multiple comparisons.

## 3. Results

**Figure 1:**
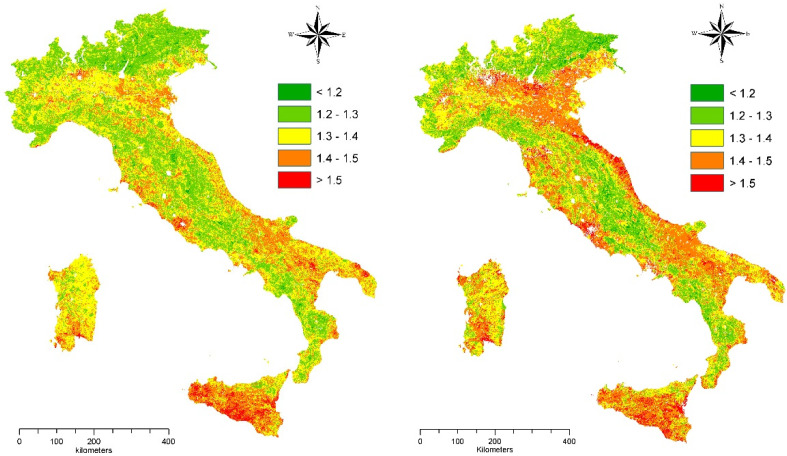
Spatial distribution of the Environmentally Sensitive Area Index (ESAI) in Italy (**left**: 1960; **right**: 2010).

A descriptive analysis of the spatial distribution of the ESAI in Italy delineated an increase of the mean scores by 1.5% (between 1.34 in 1960 and 1.36 in 2010). On average, the most sensitive provinces were observed in Southern Italy (Sicily, Sardinia, Apulia, and Basilicata). In Northern Italy, Emilia Romagna and Veneto displayed some areas with high or moderately high sensitivity scores, especially for 2010 (Figure 1).

**Figure 2:**
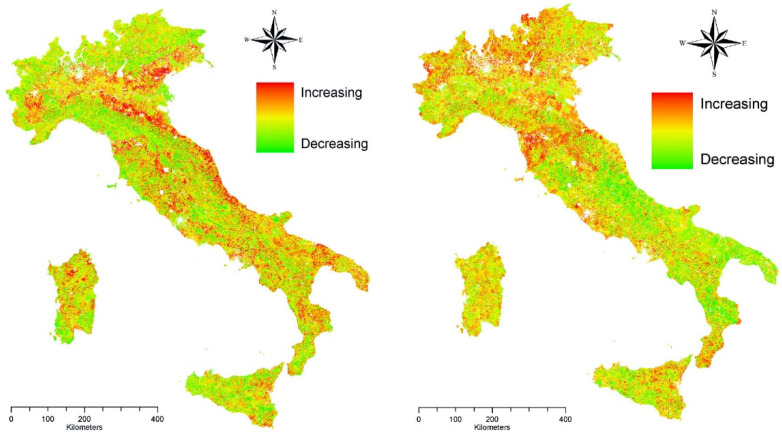
Differences over time in the Environmentally Sensitive Area Index (ESAI) in Italy (**left**: 1960–1990; **right**: 1990–2010).

The highest increase in sensitivity scores were observed along the Po River Valley, a flat district experiencing intense agricultural intensification and urban sprawl in the last decades (Figure 2). A decomposition of the increase in the ESAI score within two sub-periods (1960–1990 and 1990–2010) documents a substantially different spatial distribution that may reflect the intrinsic action of different drivers. While in the first period, vulnerability growth was more intense in already affected—or at least sensitive—areas, in the second period such increases were more evidently observed in non-affected areas, determining a sort of convergence between exposed and non-exposed land that resulted in worse environmental conditions at the base of land degradation.

Changes over time in landscape structure was investigated considering the empirical results of a Multiway Factor Analysis of metrics pooling together observations of three years (1960, 1990, 2010). Loadings of each landscape metric on the selected (principal) axes of the MFA were reported in Table 2. The first four axes (all with eigenvalues > 1) accounted for a stable proportion (more than 80%) of the explained variance in the total variance of all observation years. The first axis explained less variance over time, while the reverse pattern was observed for the second and the third axes. The temporal pattern associated with ESAI loadings was paradigmatic because it was associated specifically and uniquely with Axis 4, more intensively (0.88) in 1960, and less intensively (0.58) in 1990. Since no other metrics were associated with Axis 4 in all years (except for MNN in 2010), and assuming axes as orthogonal by construction, these findings indicate that the configuration of sensitive landscapes in each Italian province (i.e., the spatial distribution of ‘non-affected’, ‘fragile’, and ‘critical’ land) was independent from the average level of soil sensitivity (i.e., the mean ESAI score).

**Figure 3:**
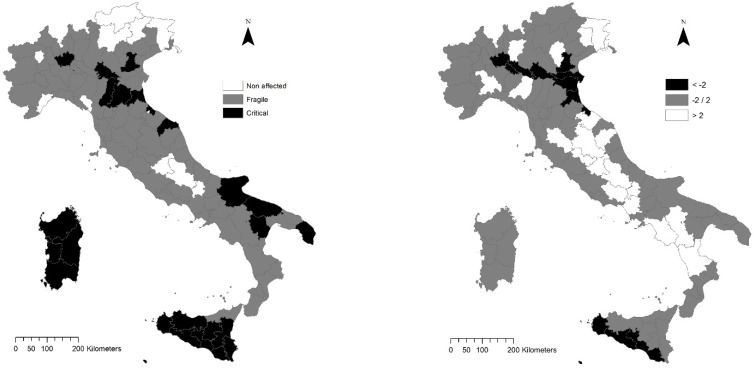
Spatial distribution of non-affected, fragile, and critical land in Italy (**left**) and factor scores (Axis 1, see Table 2) in Italy at the reference year 2010 (**right**).

In this perspective, sensitive landscapes were described using few structural dimensions in 1960: Axis 1 (43% of total variance) was associated with diversification metrics; Axis 2 was associated with shape, variability, and fractality metrics (21% of total variance); and Axis 3 (11% of total variance) was associated with fragmentation and interspersion metrics. Thirty years later (1990), landscape configuration changed moderately: Axis 1 (37%) was associated positively with diversification metrics and negatively with fragmentation metrics, Axis 2 (24%) was mostly associated with variability and fractal metrics, and Axis 3 (14%) was basically associated with mean patch size and shape metrics (positive loadings) and with location/interspersion metrics (positive and negative loadings respectively with MPI and IJI). Conversely, the results of the MFA delineated a substantial change in landscape configuration in 2010. Axis 1 (39%) represented a geographical gradient opposing landscape diversification (all diversity metrics received positive loadings) to landscape fragmentation (negative loadings assigned to MPS, MNN, and LPI). The ESAI received a negative loading to Axis 1 suggesting how fragmented landscapes were more sensitivity to degradation. In other words, an increase over time in the ESAI was associated with a process of landscape fragmentation, with the latent interspersion of ‘non-affected’, ‘fragile’, and ‘critical’ lands in a more mixed landscape matrix. Fractal dimension (Axis 2) and shape dimension (Axis 3) were basically independent from the overall degree of soil vulnerability. In this perspective, Axis 1 provides an enriched description of spatial patterns of soil sensitivity to degradation, considering not only the average ESAI alone, but integrating this composite index with other 16 landscape metrics in a (non-redundant) spatial representation of the geographical gradient from insensitive to sensitive areas in Italy. Figure 3 compares the spatial distribution of the average ESAI in Italy with the spatial distribution of Axis 1 scores derived from MFA, distinguishing Italian provinces with significantly negative scores (black) from non-significant scores (grey) and significantly positive scores (white).

This classification was based on the sign and intensity of the ESAI loading on Axis 1; since the loading received a negative sign, it means that provinces with negative scores have an above-average degree of soil sensitivity to degradation. The overall picture indicates the added value of the proposed methodology when interpreting the spatial distribution of sensitive landscapes to degradation. The global pattern is similar between the two maps: Southern areas are, on average, more sensitive than Northern areas in Italy. However, landscape analysis provided potentially richer results than the analysis of the individual ESAI score because it identified two hotspots of soil sensitivity, the former in Southern Italy (i.e., dry rural districts of Southern Sicily) and the latter in Northern Italy (i.e., the agricultural districts along the Po River in a specific district bounded by Eastern Lombardy, Southern Veneto, and Northern Romagna). These hotspots feature (i) a high degree of soil sensitivity and (ii) a moderately high landscape fragmentation over time. The resulting map supports the ESAI when focusing on practical actions against landscape fragmentation in order to assure habitat integrity, to safeguard the buffering potential of ‘non-affected’ land, and to counteract on-site and off-site soil degradation. The results of a non-parametric Kruskal–Wallis analysis of variance testing significant differences in class metrics (distinguishing ‘non-affected’ from ‘fragile’ and ‘critical’ lands) were illustrated in Table 3. In these regards, mechanization could have played an important role in the development of intensive agricultural systems, leading to fragmentation of ‘non-affected’ land patches and a synergic impact of ‘fragile’ and ‘critical’ land expanding over larger and larger areas.

The results of this inferential analysis document a progressive landscape mixing: Out of 12 class metrics, 11 metrics were significantly different among the 3 classes mentioned above in 1960, 6 metrics were significantly different in 1990, and only 2 metrics were significantly different in 2010. These findings confirm a latent process of landscape homogenization: With the expansion of sensitive areas, ‘critical’ and ‘non-affected’ land classes have progressively assumed the same structural characteristics over the whole range of Italian provinces, from North to South. Interestingly, only two metrics were stably different among land classes all over the investigated time windows: IJI (quantifying the interspersion and juxtaposition pattern of sensitive and insensitive land patches) and LSI (assessing the shape of both sensitive and insensitive land patches). ‘Fragile’ and ‘critical’ land maintained, even in more recent times, a convoluted shape and a peculiar juxtaposition pattern compared with ‘non-affected’ land. Such a differential landscape configuration suggest that shape and interspersion are relevant dimensions that may respond—more rapidly and effectively than other landscape dimensions—to specific measures preserving ‘non-affected’ land. Other dimensions—such as, for instance, patch size—became over time less effective in discriminating among ‘non-affected’, ‘fragile’, and ‘critical’ lands.

## 4. Discussion

While soil degradation and desertification indicators are regarded as useful tools for both environmental monitoring and land management, a common methodology for identification and joint application of such indicators in permanent assessment of ecological conditions is still lacking in specific world areas, such as the Mediterranean basin [31,40,45]. Changes in land cover clearly provide an important knowledge reflecting the intimate interactions between humans and nature [18,19,49]. In dry environments where fragile ecosystems are dominant, landscape transformations often reflect a significant impact on the environment due to unsustainable anthropogenic pressure [38,50,51]. While Mediterranean landscapes have been shaped by human–nature interactions over long times, the main drivers of change in such contexts remain human impact, land-use, economic development, and population growth [12,36,52]. Evaluating landscape configuration, its current state (structure) and its past/present/future changes (dynamics) provides an understanding of the ecological mechanisms and processes that drive environmental changes [5,44,53].

While desertification assessment has made increasing use of landscape ecology principles, few exercises with landscape metrics were developed with the aim at quantifying environmental change [8,27,28]. Assuming soil degradation as typically related with the spatial structure of degraded and wealthy landscapes [25,54], our study explicitly explores and integrates concepts and methodology of a landscape approach. Since spatially explicit information allow a better comprehension of ecological issues in dry ecosystems [55,56,57]. (Tao, 2004; Sun et al., 2005; Okin et al., 2009), the integration of remote sensing with Geographic Information System techniques, field surveys, and official statistics is increasingly important for the assessment of environmental problems such as soil degradation in the perspective of landscape ecology [26,51,58]. This rationale allows a refined monitoring of soil sensitivity to degradation, a reliable environmental reporting of desertification risk, and an appropriate design of containment policies based on a multidimensional indicator dashboard [30,59,60]. Starting from Italy, the proposed methodology can be easily generalized to other socioeconomic contexts in Southern Europe.

### 4.1. Landscape Dynamics and Soil Degradation

The empirical results of this study document how a landscape approach allows for a relatively quick assessment of soil degradation that can be used in supporting (regional-scale) application plans in both prevention, planning, and decision-making [25,61,62]. Considering constraints and processes that determine vegetation degradation and landscape fragmentation, human activities deteriorate landscapes more intensively around settlements and in flat districts than in strictly rural areas, reflecting the different tolerance of Mediterranean landscapes to certain degree of traditional land use [44,48,63]. Our findings also suggest that—despite being well-preserved in most cases (i.e., in non-affected land)—some risks should be more extensively considered [37], including the loss of the best-preserved vegetation patches due to human activities (e.g., shifting from ‘non-affected’ and/or ‘fragile’ land into ‘critical’ land). The comparison of landscape metrics at different levels and by means of an appropriate combination of indicators into a unifying multivariate representation allows the identification of areas with specific spatial patterns related to soil degradation, testing the validity of a landscape approach to land degradation monitoring in districts normatively (i.e., *sensu* NAP) classified as both affected and non-affected [33,34,64]. Landscape metrics set up in the present study integrate the standard approaches used in land degradation monitoring, e.g., based on individual indicators such as the ESAI [46].

Our study provides a quantitative analysis of natural and human change affecting the level of soil sensitivity to degradation in an affluent economy mentioned as partly affected in the Annex IV of the United Nations Convention to Combat Desertification (UNCCD). By delineating non-linear trends in soil sensitivity, results suggest how the spatial balance between affected and non-affected land is an important trait of any Mediterranean landscape, whose long-term equilibrium is shaped by background territorial conditions [39]. A large-scale assessment based on landscape metrics definitely illustrates—likely better than more traditional approaches—the complex shift in landscape structure and configuration [65]. In these regards, our results document how landscapes with homogeneous structures and configurations are frequently associated with high levels of soil sensitivity [35]. In other words, landscape fragmentation and diversification demonstrate to be (positive or negative) factors of soil sensitivity depending on the specific territorial context [8,9,21,23].

Using a traditional approach of landscape ecology, the intimate structure of landscapes was evaluated at three levels of soil sensitivity in Italy. With the level of soil sensitivity to degradation rising between 1960 and 2010 almost in all Italian regions [45], landscapes were proven to become increasingly fragmented, as far as the number of homogeneous patches and the mean patch size are concerned [10]. The empirical results of a multivariate analysis confirm that the increase in the level of soil sensitivity on a large scale has been associated with a structural change in the configuration of landscapes [11], altering the dynamic balance in affected and non-affected land [66]. The spatial polarization in (few, remote and unconnected) ‘critical’ land and (dominant) ‘non-affected’ land—representing the background landscape matrix in the 1960s—was mostly replaced with an intrinsically disordered landscape intermixing well-connected patches of ‘critical’ or ‘fragile’ land (expanding over time) with (smaller and more fragmented) patches of non-affected land [67,68].

Unfragmented, non-affected land represents a physical barrier to the expansion of ‘fragile’ and ‘critical’ land, i.e., acting as a buffer zone [4,69,70]. ‘Critical’ land expanded radio-centrically, incorporating both ‘fragile’ and ‘non-affected’ areas and forming a structured spatial network [71]. ‘Non-affected’ land has been strongly fragmented, acting less effectively as a buffer to the expansion of ‘critical’ land [6]. Displaying a spatially additive expansion, ‘fragile’ land has in turn undergone fragmentation processes [13]. Spatial polarization in affected and non-affected areas was progressively more intense, especially in Southern Italy, often resulting in a fractal landscape [30,52,72].

Provinces exposed to a higher level of soil sensitivity featured a more homogeneous landscape [73], with increases in the average size of ‘critical’ land patches and uneven fragmentation of non-affected land patches that may erode their capacity to buffer further vulnerability increases and to resist to external shocks leading to renewed processes of land degradation [48]. In these regards, the recent spatial diffusion of patches exposed to intrinsic degradation (i.e., ‘critical’ land, considered as ‘hotspots’ of soil degradation) may bring to important consequences in two directions [74]. The increasing number of hotspots may leverage the intrinsic probability of local-scale soil degradation. At the same time, since the buffering effect of neighboring (non-affected) land is supposed to be more effective on smaller (than larger) hotspots, this phenomenon may bring a self-alimenting expansion of sensitive soils [75]. This result appears particularly relevant for ‘zero net’ land degradation strategies, since preserving the spatial integrity and connectivity of non-affected land is an important planning tool containing the risk of desertification [47]. At the same time, acting preventively on the landscape mechanisms that stimulate the radio-centric, self-additive expansion of ‘critical’ and ‘fragile’ lands and reducing the connectivity of non-affected land, appear a reasonable measure reinforcing the adaptation of local landscapes to external disturbances, e.g., climate change, urbanization, and crop intensification [76]. Proactive policies enforcing a more effective protection of relict unfragmented land could successfully reverse the trend of growing landscape fragmentation and fractalization.

### 4.2. Policy Implications

The present study finally documents how the spatial balance between ‘critical’ and ‘non-affected’ land is a particularly important trait of any Mediterranean landscape, whose dynamic equilibrium is strongly influenced by the background territorial (i.e., socioeconomic and environmental) conditions [66]. In this direction, landscape metrics are refined indicators of soil sensitivity [60] and provide an information dashboard of (apparent and latent) landscape dynamics [59]. The analysis run on a provincial scale also allows for an operational use of these indicators from an integrated policy perspective. In Italy, the National Action Plan against Desertification (NAP) provides for (and coordinates) the implementation of Regional Action Plans (RAPs), which can largely benefit from the quantitative information presented in our work. In particular, landscape metrics offer a multivariate reading of vulnerable landscapes [77], going beyond the unidimensional indications provided with the ESAI ranking [49].

The multivariate analysis of landscape metrics run in this study indicates how landscape structure was highly diversified at the provincial level in Italy [38]. In all study periods, the empirical results of the analysis go beyond the traditional dichotomy between Northern (non-affected) and Southern (affected) regions, highlighting a more heterogeneous spatial framework that mixes soil/land classes as a function of changes in the dominant landscape [31]. These results outline highly differentiated levels of soil sensitivity in both regions. In this view, concentrating efforts to mitigate and adapt to the risk of desertification in affected areas of Southern Italy—intended as the general strategy of the NAP—should be, at least in part, reconsidered. Based on landscape metrics and their empirical relationship with the ESAI, some provinces of both Southern Italy (Sardinia, Sicily, Puglia) and Northern Italy (Emilia Romagna, Veneto) share high levels of soil sensitivity to degradation and, therefore, they can benefit from specific strategies of mitigation and adaptation [43]. Reconsidering territorial classifications of affected and non-affected areas, acquiring more information at a disaggregated spatial level, and evaluating together structure, composition, and functions of ‘non-affected’, ‘fragile’, and ‘critical’ lands are urgent tasks of any policy strategy oriented toward ‘zero-net’ land degradation [22,78,79]. Landscape metrics provided a suite of indicators ancillary to the ESAI, giving a complete overview of the relationship between soil sensitivity to degradation and landscape fragmentation [46]. In line with these results, future implementations may consist of, e.g., generating maps of agricultural mechanization and other sensitivity indicators for wider areas.

The research design performed in this study exploited a multivariate framework based on sequential statistical steps. Distinguishing landscape dynamics and trends of each individual class, an exploratory analysis provided the appropriate assessment of the relationship between soil sensitivity and land transformations [18,20,41,80]. Redundancy among landscape metrics was treated using a factor analysis that decomposed indicators into few independent (i.e., non-redundant) dimensions relevant to the analysis of land degradation trends. A non-parametric analysis of variance assured an additional verification of significant differences in each individual metric across soil sensitivity classes, i.e., comparing potentially affected, fragile, and critical land and identifying peculiar (structural and functional) traits of the related landscape [3,35,81]. The results of this approach informed strategies of sustainable land management based on a comprehensive investigation of landscape configuration as far as the different degrees of sensitivity are concerned [10,82,83]. The results of these analyses were found to be in line with the predictions of the Italian National Action Plan and the implementation of many Regional Action Plans to combat desertification. Both documents evidence the need of a suite dashboard of landscape indicators delineating the multiple dimensions of soil sensitivity to degradation over time and space [2].

## 5. Conclusions

The use of traditional landscape metrics applied to soil degradation maps contributes to permanent monitoring of desertification risk. These metrics provide detailed information on changes over time in the structure and composition of land with a different degree of sensitivity (‘non-affected’, ‘fragile’, ‘critical’), thus representing a reliable early-warning dashboard of desertification indicators. Such indicators substantiate and enrich the information related to the average level of sensitivity (landscape scale) and associated with the ESAI. The integrated analysis of these metrics based on policy-relevant spatial domains shed light on degradation dynamics, linking (at least indirectly) landscape transformations with the underlying socioeconomic context. This logical scheme justifies the adoption of an administrative spatial scale (i.e., province) for the environmental reporting of landscape metrics related to the ESAI land classification system. This spatial level is aggregated enough to (i) contain a wide range of change processes, (ii) represent the relevant socioeconomic dynamics on a territorial scale, and (iii) reflect an operational dimension of policy against the risk of desertification, as suggested in the Italian NAP, which delegates local-scale containment measures to the respective Regional Action Plans. Based on these premises, our work has definitely shown how the availability of large datasets with diachronic information allows a more comprehensive vision of the intimate landscape transformations at the base of soil degradation. This knowledge supports the formulation of targeted, place-specific planning actions counteracting the risk of desertification. Technological challenge and the growing interest in open data provides an information base of interest in this direction. At the same time, it appears increasingly necessary to make available diachronic information (e.g., from reliable data sources such as historical land-use maps) that allow a long-term assessment of landscape dynamics.

## Figures and Tables

**Table 1:** List of landscape metrics used to study the spatial distribution of land vulnerability to degradation in Italy.

Acronym	Metric	Rationale
MPI	Mean proximity index	The degree of isolation and fragmentation of the corresponding patch type
MNN	Mean nearest neighbor distance	The shortest straight-line distance between the focal patch and its nearest neighbor of the same class
IJI	Interspersion/juxtaposition index	The observed interspersion divided by maximum possible interspersion for the given number of patch types
MPS	Mean patch size	The arithmetic mean of the patch sizes
PSCoV	MPS coefficient of variation	The coefficient of variation in patch size relative to the mean patch size
ED	Edge density	The sum of the lengths of all edge segments, divided by the total area
MSI	Mean shape index	The average perimeter-to-area ratio for weighted by the size of its patches
AWMSI	Area-weighted mean shape index	The average shape index of patches, weighted by patch area
MPFD	Mean patch fractal dimension	The sum of 2 times the logarithm of patch perimeter divided by the logarithm of patch area for each patch of the corresponding patch type, divided by the number of patches of the same type
AWMPFD	Area-weighted mean fractal dim.	The average patch fractal dimension, weighted by patch area
LPI	Largest patch index	The percent of the landscape or class that the largest patch comprises
LSI	Landscape shape index	The sum of the landscape boundary and all edge segments within the landscape boundary divided by the square root of the total landscape area
SDI	Shannon diversity index	Minus the sum, across all patch types, of the proportional abundance of each patch type multiplied by that proportion
SHEI	Shannon evenness index	The observed Shannon’s Diversity Index divided by the maximum Shannon’s Diversity Index for that number of patch types
SIEI	Simpson’s evenness index	The observed Simpson’s Diversity Index divided by the maximum Simpson’s Diversity Index for that number of patch types
MSIEI	Modified Simpson’s even. Index	The observed modified Simpson’s diversity index divided by the maximum modified Simpson’s diversity index for that number of patch types

**Table 2:** Results of a Multiway Factor Analysis run on the full set of landscape metrics (see Table 1 for acronyms) considered in this study at the provincial scale in Italy, by year; only significant loadings were reported here.

Metric	1960				1990				2010			
	Axis1	Axis2	Axis3	Axis4	Axis1	Axis2	Axis3	Axis4	Axis1	Axis2	Axis3	Axis4
MPI	0.64					0.69	0.55			0.60	0.57	
MNN	−0.61								−0.63			0.65
IJI			0.69		0.58		−0.68		0.57		−0.62	
MPS			−0.85		−0.53		0.69		−0.62			
PSCoV		0.91				0.80				0.80		
ED	0.60	−0.59			0.66					−0.62		
MSI	0.76				0.50		0.74				0.88	
AWMSI	0.61	0.75				0.96				0.88		
MPFD	0.62						0.56				0.71	
AWMPFD	0.67	0.66				0.93				0.85		
LPI	−0.74				−0.87				−0.77			
LSI	0.72	0.58				0.82			0.64	0.63		
SDI	0.81				0.94				0.96			
SHEI	0.89				0.94				0.94			
SIEI	0.96				0.95				0.94			
MSIEI	0.93				0.94				0.93			
ESAI				0.88				0.58	−0.57			
Variance (%)	43.4	20.6	11.1	6.0	36.8	24.1	13.8	6.4	38.7	21.5	17.4	5.8

**Table 3:** Results of a non-parametric Kruskal–Wallis analysis of variance (*z*-score) testing significant (*) differences (*p* < 0.05 after Bonferroni’s correction for multiple comparisons) in selected metrics among the three ESAI classes (non-affected, fragile, critical) in Italy, by year (landscape diversity metrics were not calculated at the class scale).

Metric	1960	1990	2010
MPI	6.3 *	0.8	0.6
MNN	7.1 *	0.8	0.1
IJI	7.6 *	6.3 *	6.6 *
MPS	8.0 *	1.4	0.3
PSCoV	0.1	0.3	0.4
ED	5.8 *	4.3 *	2.3
MSI	7.6 *	3.4 *	2.0
AWMSI	6.2 *	2.9 *	1.9
MPFD	5.6 *	1.1	1.1
AWMPFD	6.4 *	3.2 *	2.3
LPI	7.8 *	0.3	0.8
LSI	2.9 *	4.3 *	3.8 *

## Data Availability

Official statistics from public data sources were only used in this study.

## Supplementary Materials

The following supporting information can be downloaded at: https://www.mdpi.com/article/10.3390/ijerph19052710/s1, File S1: The ESA methodology.
