# Supplementary material for: Found in Complexity, Lost in Fragmentation: Putting Soil Degradation in a Landscape Ecology Perspective
Source: Int J Environ Res Public Health. 2022 Feb 25;19(5):2710. doi: 10.3390/ijerph19052710 (PMC8910665; doi:10.3390/ijerph19052710)
Supplement: Supplementary file 1 [file ijerph-19-02710-s001.zip › ijerph-1530556-supplementary.pdf]

Supplementary File S1

The ESA methodology

The Environmentally Sensitive Area (ESA) approach was launched in 1987 in the UK by the Ministry of Agriculture, Fisheries and Food (now the Department for Environment, Food and Rural Affairs) to encourage farmers and landowners to adopt environmentally-friendly land management practices (Wilson, 1996). In the early 1990s, the ESA framework was adapted to monitor desertification processes on the behalf of the MEDALUS project (Kosmas *et al.*, 1999). Although possible drawbacks of this framework have been discussed by Basso *et al.* (2000, 2012) and Bajocco *et al.* (2011), the ESA scheme remains one of the most well-used procedures to evaluate the sensitivity of land to desertification (e.g. Kosmas *et al.*, 1999; Bakra *et al.*, 2012; Mohammed, 2012). The main advantages of the ESA are flexibility in the use of the input variables and the simplicity of the land classification based on its level of sensitivity. The outcomes of the ESA model have been extensively validated on the ground at several sites in southern Europe (Kosmas *et al.*, 1999; Basso *et al.*, 2000; Bajocco *et al.*, 2011) and a regional assessment (Lavado Contador *et al.*, 2009) based on heterogeneous geographical datasets with different reliability, indicates the ESAI as a proxy for land degradation processes and identifies significant correlations with a number of indicators of soil degradation. Finally, Ferrara *et al.* (2012) evaluated the stability of the ESAI using statistical analysis and the sensitivity to changes in the indicators. Results indicate that the ESAI is a stable and reliable index not significantly affected by spatial and temporal heterogeneity in the composing indicators.

Despite its acknowledged importance as a tool to detect desertification risk, the ESA approach presents some shortcomings (e.g. Salvati *et al.*, 2013). The methodology does not provide an assessment of the importance of the individual variables or thematic indicators. In addition, the input variables are oriented towards the description of the bio-physical conditions of the area, while a number of socio-political and cultural factors considered as important in influencing the processes of land degradation, is not explicitly formalized through the use of appropriate quantitative variables (Salvati & Bajocco, 2011).

According to the ESA framework the variables selected to study the level of land sensitivity to desertification in Italy refer to four themes: climate quality, soil quality, vegetation and land use quality, and human pressure/land management quality (Table I). In our experience, the layers used are the most reliable, updated and referenced data currently available to be used in the regional and country assessment of the ESAI in Mediterranean countries (see also Salvati, 2012 for a discussion on supply-demand of statistical data in desertification matters). Since comparable data, needed to develop the full ESAI model (*sensu* Salvati & Bajocco, 2011) with national coverage and detailed spatial scale were available only at limited dates (see Table II), we covered a time period encompassing fifty years by specifically investigating the level of land sensitivity in four specific years (1960, 1990, 2000 and 2010) and providing an estimate for selected variables in three specific years (1970, 1980 and 2005). In particular, while climate and human pressure variables were observed at each of the seven points in time, vegetation variables were observed at four years and estimated for the three remaining years.

Environmental variables and thematic indicators

Climate quality has been described in the present study using the following variables: average annual rainfall rate, aridity index, and aspect (Basso *et al.*, 2000). Rainfall rate and the aridity index were calculated on a ten-year base using information collected in the Agro-meteorological Database of the Italian Ministry of Agriculture. The database relates to gauging data collected daily from various meteorological and hydrological networks (Italian Ministry of Agriculture, National Hydrological Service, Italian Air Force, and some minor networks) operating with nearly 3,000 weather stations since 1951. The aridity index was defined as the ratio between rainfall and reference evapotranspiration measured as a ten-year average. The reference evapotranspiration rate was calculated by using the Penman-Monteith formula (Salvati & Bajocco, 2011). Aspect was derived from elaboration on the ASTER (Advanced Spaceborne Thermal Emission and Reflection Radiometer) global Digital Elevation Model (DEM) at 30m resolution scale generated from stereoscopic pairs of optical ASTER images and freely available online at http://www.gdem.aster.ersdac.or.jp/. Meteorological data were interpolated through geo-statistical procedures (using elevation, latitude, and distance to the sea as ancillary variables) to ensure the homogeneous national coverage. A grid composed by 544 points with daily data of temperature, precipitation, humidity, solar radiation, and wind has been created. Seven analysis periods were selected: 1951-1960, 1961-1970, 1971-1980, 1981-1990, 1991-2000, 1996-2005 and 2001-2010.

Soil data derived from the European Soil Database at a 1 km^2 pixel resolution (Joint Research Centre, JRC). The following sources of data also provided ancillary information: (i) an Italian database of soil characteristics ('Map of the water capacity in agricultural soils') generated by the Ministry of Agriculture and based on nearly 18,000 soil samples (Salvati, 2012); (ii) thematic cartographies including Ecopedological and Geological maps of Italy, obtained from the Joint Research Centre and the Italian Geological Service) and, finally, (iii) a land system map produced by the National Centre of Pedological Cartography. These datasets can be considered as the standard, homogeneous soil information available in Italy at 1:250,000 scale. The variables considered in this study include soil depth and texture, slope, and the nature of the parent material. These variables can be considered as proxy information for other soil quality indicators (e.g. organic matter content, resistance or tendency to compaction). Soil structural characteristics including texture, depth, and parent material are determined by the joint action of factors including climate, soil organisms, morphology, and time (Kosmas *et al.*, 1999). In our case study, considering the examined time span, these variables have been regarded as static during the study period because they change slowly, if at all or, by their nature, are infrequently measured (Bajocco *et al.*, 2011). The long investigated time period and the national coverage of the study prevented us from using diachronic soil mapping available at the very local scale. However, it should be noted that, among the considered variables, soil depth can vary along prolonged time intervals and in places with specific territorial characteristics possibly due to the effect of soil erosion.

The importance of vegetation cover in land degradation processes was evaluated through four variables:

vegetation cover, fire risk, protection offered by vegetation against soil erosion, and the degree of resistance to drought shown by vegetation (Basso *et al.*, 2000). Such variables derived from elaboration on two comparable maps: the CORINE-like 'Topographic and Land Cover Map of Italy' (Colamonico, 1971) produced by the National Research Council and the Italian Touring Club in 1960 (LUM60), and three CORINE land cover maps respectively dated 1990 (CLC90), 2000 (CLC00), and 2006 (CLC06). Variables were determined by applying a weighting system (ranging from 1 to 2 and derived from Kosmas *et al.*, 2000) that classifies each observed land cover class according to the level of sensitivity to land degradation. The LUM60 is a standard map covering the whole Italian territory at 1: 200,000 scale and classifying land cover in 22 categories according to a nomenclature which is compatible with the CLC hierarchical system (Falcucci *et al.*, 2007). Based on topographic maps provided by the Italian Touring Club and the Italian Geographical Military Institute and dated 1949-1962, the LUM60 map was prepared integrating cadastral maps, an extensive field survey together with statistical data at a fine spatial scale. The map was already used for diachronic comparisons with the CLC cartography (Falcucci *et al.*, 2007) and for multi-temporal analysis of land cover and other environmental indicators (Salvati, 2012). The CLC program was developed by the European Environment Agency (EEA) using satellite imagery to provide pan-European, diachronic 1:100.000 land cover maps with 25 ha minimum mapping unit. The CLC nomenclature includes 44 land cover classes grouped into a three-level hierarchy.

Due to the lack of comparable land cover maps covering the whole national territory at three years (1970, 1980 and 2010), LUM60 and CLC90 were used to estimate the Vegetation Quality Index (VQI) respectively in 1970 and 1980, whilst CLC06 was used to estimate the VQI in 2010. Although the data material used in the present study has obvious shortcomings, this may be acceptable when the purpose is to study a large region (e.g. a whole country) over a long time interval, since the cost of mapping is insurmountable for an individual research project. It is therefore inevitable that such large scale studies rely on sources of varying accuracy.

Anthropogenic pressure and land management quality which can cause land degradation processes have been quantified as the result of population dynamics and selected land-use changes (Otto *et al.*, 2007). Density and annual growth rate of resident population have been used as proxy indicators of human pressure. Demographic density was assessed at the municipal scale in 1961, 1971, 1981, 1991, 2001, 2006, and 2011 on the basis of the National Censuses of Population and the annual Population Register held by the Italian National Institute of Statistics (ISTAT, 2006). Population increase (or decrease) was determined as the annual demographic change observed at the same spatial scale in the following period: 1951-1961, 1961-1971, 1971-1981, 1981-1991, 1991-2001, 2002-2006, 2007-2011. Finally, an indicator of land-use intensity was obtained by applying a weighting system (ranging from 1 to 2 and derived from Salvati & Bajocco, 2011) that classifies the observed classes according to their intensity of use and potential level of sensitivity to degradation. This indicator was obtained from elaboration on the maps previously cited (LUM60 and CLC90, CLC00, and CLC06).

The composite index of land sensitivity to degradation

The ESAI framework quantifies sensitivity to land sensitivity as a combination of unsustainable land management together with environmental factors including poor soil, vegetation cover and dry (or drier) climate (Basso et al., 2000; Lavado Contador *et al.*, 2009). A scoring system is applied, based on the known relationship between each factors and land degradation processes. The weighting system suggested by Salvati & Bajocco (2011) was adopted in the present study. This system followed the benchmarking system introduced by Kosmas *et al.* (1999), Basso *et al.* (2000), and Lavado Contador *et al.* (2009). The ESA framework produces quality indicators of climate (Climate Quality Index, CQI), soil (Soil Quality Index, SQI), vegetation (Vegetation Quality Index, VQI), and land management (Land Management Quality Index, MQI) that are estimated as the geometric mean of the different scores assigned to each input variable. Each indicator ranges from 1 (the lowest contribution to land sensitivity to degradation) to 2 (the highest contribution to land sensitivity to degradation). The ESAI was then estimated in each spatial unit and year as the geometric mean of the four quality indicators (CQI, SQI, VQI, MQI) obtaining a score ranging from 1 (the lowest sensitivity to degradation) to 2 (the highest sensitivity to degradation). The four indicators weighted the same in the ESAI procedure (Kosmas *et al.*, 1999). Four classes of land sensitivity were identified that reflect the classification threshold shown in Salvati & Bajocco (2011): (i) areas unaffected by LD (ESAI < 1.17), (ii) areas potentially affected by LD (1.17 < ESAI < 1.225), (iii) 'fragile' areas (1.225 < ESAI < 1.375), and (iv) 'critical' areas (ESAI > 1.375). Maps have been produced at 1 km^2 pixel resolution (Salvati, 2012). The elementary spatial unit has been selected according to Basso *et al.* (2000) and is coherent with the resolution of the single layers.
